# Do we harm others even if we don't need to?

**DOI:** 10.3389/fpsyg.2015.00729

**Published:** 2015-06-02

**Authors:** M. Paula Cacault, Lorenz Goette, Rafael Lalive, Mathias Thoenig

**Affiliations:** Department of Economics, University of LausanneLausanne, Switzerland

**Keywords:** parochial altruism, experimental tests, public-good, in-group favoritism, out-group aggression, strong aggression

## Abstract

Evolutionary explanations of the co-existence of large-scale cooperation and warfare in human societies rest on the hypothesis of parochial altruism, the view that in-group pro-sociality and out-group anti-sociality have co-evolved. We designed an experiment that allows subjects to freely choose between actions that are purely pro-social, purely anti-social, or a combination of the two. We present behavioral evidence on the existence of *strong aggression*—a pattern of non-strategic behaviors that are welfare-reducing for all individuals (i.e., victims and perpetrators). We also show how strong aggression serves to dynamically stabilize in-group pro-sociality.

## 1. Introduction

“…Ich bin ein Teil von jener Kraft, die stets das Böse will, und stets das Gute schafft” (*Faust*, 1335–1336).

[I am part of that power which eternally wills evil and eternally works good].

Human societies are unique in the animal world. They are characterized by extensive cooperation in large groups of genetically unrelated individuals (Fehr and Fischbacher, 2003; Boyd and Richerson, 2006). But they are also ravaged by intergroup cleavages and conflicts with huge death toll arising due to genocides, ethnic cleansing, terrorism, and wars in modern societies (Doyle and Sambanis, 2006; Blattman and Miguel, 2010). Ethnographic research suggests that warfare was a leading cause of death and presumably an important driver of the evolution of Homo Sapiens in foraging societies (Chagnon, 1988; Keeley, 1996; Bowles, 2006; Gat, 2006). This dual aspect of human social behavior has been an enduring puzzle in evolutionary biology (Darwin, 1871; Hamilton, 1975), in social sciences (Baron, 2001; Posner, 2004; Sambanis et al., 2012) and in humanities as illustrated by our quote from Mephistopheles' famous statement to Faust (von Goethe, 1808). A prominent evolutionary explanation of this joint phenomenon rests on the hypothesis of parochial altruism (Wilson, 1975; Avilés, 2002; Boyd et al., 2003; Franck, 2003; Bowles, 2009). Though a catch-all term, we define parochial altruism as the set of behaviors and heuristics combining strong forms of in-group pro-sociality and out-group anti-sociality that translate into actions where individuals, at *personal cost*, help members of their in-group while also hurting others in the out-group. Thus, violence and atrocities are often perpetrated in the name of group interests and history abounds with tales of self-sacrifice in war. For instance, Arnold von Winkelried, a Swiss soldier, is reputed to have helped the Swiss to victory over the army of the Habsburg regime in the battle of Sempach in 1386. He saved the battle by throwing himself into the Austrian attack formation, grabbing as many spears as he could and having them impale him. He fell dead but his action opened a hole in the attack formation and helped his compatriots to rush to victory. Winkelried personifies an extreme combination of in-group cooperation and inter-group hostility.

In this paper, we design incentivized experiments to test the key set of phenotypic predictions at the core of the theory of parochial altruism. We first test for the existence of *strong aggression*: a pattern of antisocial behaviors which are designed to reduce the welfare of the out-group, do not improve welfare for the in-group compared to other peaceful behaviors, and are not motivated by cross-group strategic concerns (e.g., retaliation or preventive strike). Secondly, we ask whether the presence of aggression increases cooperation with the in-group. Theories of parochial altruism (Choi and Bowles, 2007; Lehmann and Feldman, 2008; Lehmann, 2011) suggest that we should observe the aggressive trait not in isolation but rather systematically associated to its in-group pro-social counterpart. We are able to identify this relationship because our experimental design allows participants to either be cooperative with their in-group, hurt the out-group, or cooperate with their in-group and hurt the out-group at the same time. Thirdly, the parochial theory claims that, in human evolutionary history, the stabilization of in-group pro-sociality has been ensured by the simultaneous emergence of out-group aggression. We study the dynamics of in-group cooperation in a repeated interaction setup and assess whether the mere existence of anti-social actions dynamically stabilizes in-group cooperation, even when repeated-game incentives vanish.

Research on parochial altruism has focused on the theoretical channels through which selective pressures have calibrated human brain and cognition—i.e., genetic evolution in small groups of hunter-gatherers or cultural evolution in large groups with norm enforcement and social punishment (Henrich et al., 2006, 2010; Choi and Bowles, 2007; Lehmann and Feldman, 2008; Bell et al., 2009; Lehmann, 2011; Mathew and Boyd, 2011). However, phenotypic evidence on parochial altruism is scant. Some studies show that anti-social motivations are quite common in many societies and organizations (Abbink et al., 2010; Goette et al., 2012). Several behavioral investigations document the in-group bias of pro-sociality and its moderation by group salience (Goette et al., 2006; Efferson et al., 2008; Yamagishi and Mifune, 2009; Abbink et al., 2010; Alexander and Fotini, 2011). These papers look at how cooperation is affected by in-group vs out-group membership and test for the presence of “in-group love” and “out-group hate” using manipulations of group membership (e.g., minimal groups or private/public knowledge about group membership). Our study complements the literature by providing evidence for strong aggression. Strong aggression differs from out-group hate that is defined as a “reduced level of cooperation with out-group members” (Yamagishi and Mifune, 2009, p. 230). In our design group-salience is not manipulated and out-group hate is muted as subjects are not allowed to cooperate with their out-group in any way.

Existing evidence in the literature is consistent with parochial altruism but it discusses only partial and indirect manifestations of parochialism. The crucial aspect of parochial altruism is the complementarity between in-group pro-sociality and out-group anti-sociality. With this respect, our study differs in important ways from earlier work on aggression in the laboratory. In our design participants can reach any given level of in-group cooperation without exerting out-group aggressive behaviors^1^. The strategic proximate motives to display out-group aggression are thereby muted and aggression is not a necessary precondition for achieving in-group cooperation as in earlier work (Bornstein and Ben-Yossef, 1994; Tan and Bolle, 2007). Moreover, in our design, strong aggression is elicited in an asymmetric way such that there is no overlap between perpetrating groups and victimized groups. This feature is the key difference with the intergroup prisoner's dilemma-maximizing differences game (IPD-MD, Halevy et al. 2008; De Dreu et al. 2010). In particular, an important result by Halevy et al. (2008) is that participants to the IPD-MD do not exhibit strong aggression and exert peaceful cooperation when they are allowed to. We hypothesize that this pattern is due to the symmetric design of the IPD-MD that presumably promotes peaceful behaviors: Expectations of retaliation by the other group could potentially inhibit aggressive behaviors. By contrast, those expectations play no role in our asymmetric version of the IPD-MD and our results clearly show that participants exhibit patterns of strong aggression in this asymmetric setup. Zizzo (2004) discusses an experiment where, by paying a price, subjects could then eliminate (burn) and redistribute money (including their own) and, in about half of the sessions, steal money from others. He finds that about 20% of the subjects “burn” money and stealing also occurs quite frequently. There is a key difference between this experiment and ours: while burning money reduces inequality in Zizzo (2004)'s setting, the corresponding options in our design increased inequality.

## 2. Materials and methods

We implement the test for parochial altruism in the context of a linear Public Good laboratory experiment. Participants were recruited from a subject pool composed mainly of undergraduate students at the University of Lausanne (UNIL) and at the Federal Polytechnic School of Lausanne. Invitations were sent to a random subset of the subject pool; we excluded psychology students because they may have been participating in experiments that involve deception. None of the conditions implemented in this experiment involved deception and the experimental procedure was approved by the Ethics' Committee of the Faculty of Business and Economics (HEC) of the UNIL. All experiments were anonymous, computer-mediated (Fischbacher, 2007), with a strict enforcement of non-communication between participants, and the instructions were phrased in a neutral way (with no reference to aggression, competition, victimization, etc. C.f. Section 2 of the Supplementary Material for a translation of the instructions). Upon arrival at the meeting point, participants signed a consent form in line with the aforementioned ethics' committee guidelines.

We organized 8 sessions gathering 18 participants each, who were randomly assigned to a three-person group (their *in-group*). Each group was exposed to one, and only one, of the three following conditions (see Table 1): The control condition is a standard linear public good game; aggression is a treatment condition and corresponds to a variant of the public good game where members of this group can perpetrate aggression against a victimized group; victimization is a treatment condition where group members are engaged into a standard linear public good game and simultaneously are victimized by another group. Groups were randomly assigned to the control, aggression or victimization condition and there was no strategic interaction between these groups whatsoever. Group membership was reinforced by a minimal-group manipulation with shirts of different colors, depending on the condition (Control: orange, Aggression: green, Victimization: blue). We neither manipulate the emotional state of individuals, nor do we induce inter-group hostility, nor do we manipulate information on group membership. Our key manipulation concerns the actions individuals can take and the effects of their actions for others.

**Table 1:** **Summary of the options available to participants in the different conditions**.

**Condition**	**Control**	**Victimization**	**Aggression**
Available options	Keep	Keep	Keep
	Project A	Project A	Project A
			Project B
			Project C
**DESCRIPTION OF THE OPTIONS**			
Keep:	1 MU invested in this option yields 1 MU to the individual, i.e., players keep the MUs in their individual account.			
Project A:	1 MU invested yields 2 MU that are distributed equally among the three group members. The extra MU comes from the Experimenter.			
Project B:	1 MU invested yields 2 MU that are distributed equally among the three group members. The extra MU comes from the Victimized group.			
Project C:	1 MU invested yields 0 MU. 1 MU is deducted from the Victimized group.			
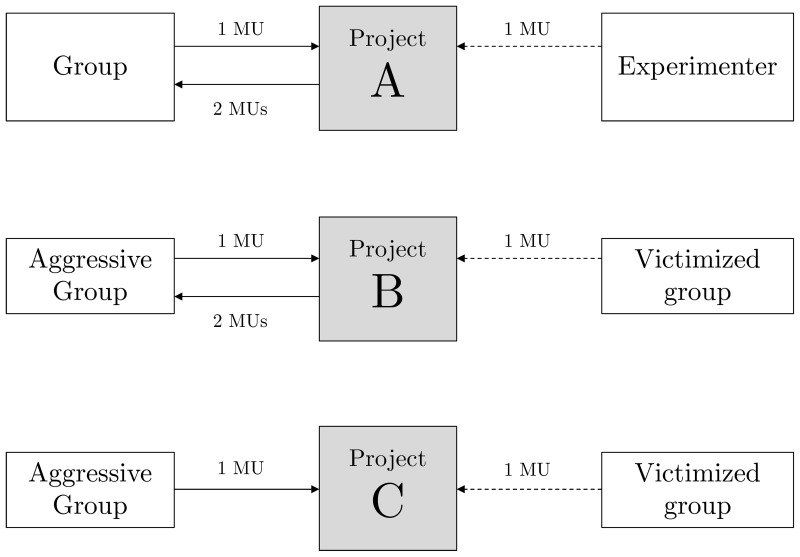			

In the control condition, members of each group received an individual endowment of 30 monetary units (MUs) that they were entitled to keep or to allocate partially/totally to an in-group pool called “Project A.” Every MU contributed to this in-group pool is doubled (by the experimenter) and shared equally among the three in-group members. The game was played for six periods with the same group composition. After each period, participants stated their beliefs about the contribution to non-selfish option A of the other individuals in their in-group. Since the group composition was stable, there was the scope for cooperation early in the game due to incentives stemming from finite repetition. In this condition, we expect the standard pattern of initial cooperation to steadily decline over periods as players approach the final period of the public good game (end-game effect).

In the aggression condition, participants could also keep or partially/totally allocate their endowment to Project A, but two additional options were made available to them. The first option is an in-group pool called “Project B” where every MU is doubled and shared equally among the in-group members. In contrast to project A, the extra MU in project B comes from the victimized group reducing its payoff by one MU. The second option is “Project C,” where every MU contributed to that option is lost to members of the in-group and it reduces the payoff of the victimized group by one MU. The game was played for six periods. After each period, participants saw the total number of MUs invested in projects A, B, and C and by how much the payoff of the victimized group was reduced as a result of contributions to B and C. Subjects then stated their beliefs about the average contribution to non-selfish options A, B, and C of the other individuals in their in-group. Notice that contributions to projects A and B generate identical payoffs for the in-group. The key difference is that project B entails aggression toward the out-group whereas project A is neutral to the out-group. Contributions to project B and C are consistent with *strong aggression*, as they reduce out-group welfare, do not improve in-group welfare compared to project A, and can not be motivated by retaliation.

In the victimization condition, members of the group play the standard public good game. In contrast to the control condition, payoffs received by the victimization participants are reduced in a way defined by decisions of the assigned perpetrating group. Importantly these reductions are independent of decisions of members of the victimized group. Consequently, in-group cooperation does not allow victimized group members to shield against external aggression.

Notice that in all three conditions contributions are affected by the standard social dilemma: not contributing yields the highest individual payoff regardless of what others do.

At the end of each session, participants completed an open-answer survey with questions on why they chose to contribute to the projects available to them. We make use of this information on motivational aspects in order to complement the behavioral evidence on strong aggression.

## 3. Results

**Figure 1:**
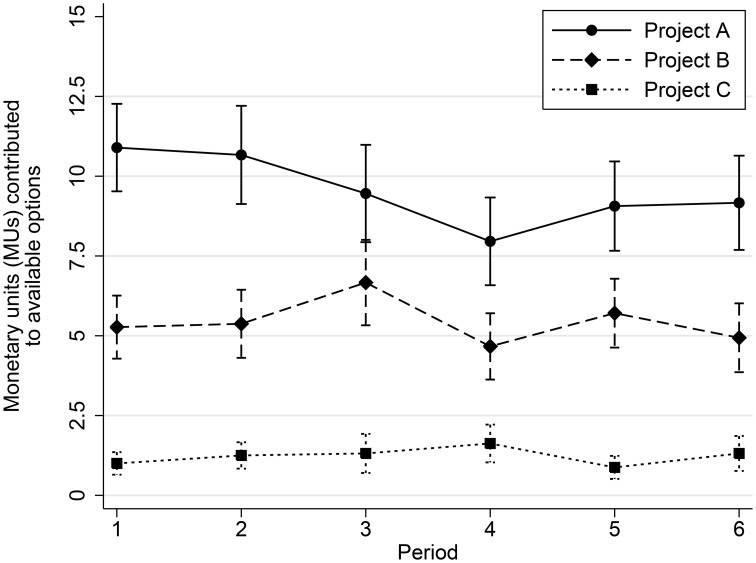
**This figure shows the average number of monetary units (MUs) contributed to project A, project B, and project C (mean ± 1 standard error) among the individuals in the aggression condition (see Table 1 for description of the projects)**. Contributions to project A are significantly higher than contributions to project B (two-sample mean-comparison test (paired) *t* = 4.78, *p* < 0.01) but individuals also contribute to anti-social projects B and C—options that reduce the payoffs to individuals in the victimized group.

Our first result is that antisocial projects B and C are used throughout the game in the aggression condition (see Figure 1). Participants contribute around 9 out of 30 MUs to the purely cooperative project A, around 5 MUs to the anti-social project B, and about 1 MU to the anti-social project C. Anti-social projects (B and C) make up 30–40% of total non-selfish contributions (projects A, B, and C) and this amount is statistically significant (one-sample mean-comparison test *t* = 12.87, *p* < 0.01). Contributions to the purely anti-social project C are positive (one-sample mean-comparison test *t* = 6.16, *p* < 0.01), but significantly lower than contributions to project B, which is both anti-social to the out-group and pro-social to the in-group (two-sample mean-comparison test *t* = 9.11, *p* < 0.01, c.f. Figure 1). This evidence suggests that purely aggressive behavior is rare, but it becomes much more prevalent if it can be combined with in-group pro-sociality.

Participants who invest into projects B and C display behaviors consistent with *strong aggression*. However, there are two alternative motivational drivers that we would like to discuss: Firstly, participants could contribute to B rather than A because they are indifferent to the consequences on the victimized group; secondly, participants who contribute to C could have been merely confused and chosen this option by mistake. We investigate this issue by analyzing answers to the survey on why participants in the aggression condition contributed to projects A, B, and C. Table 2 displays the list of motives for each project, by order of frequency (see Section 3 of the Supplementary Material for details about this classification and examples of participants' statements). Results show that participants who contributed to A did so to increase earnings of the group (15 out of 44) or increase earnings of the group without harming the other group (13 out of 44 subjects). This evidence suggests that most subjects in the lab were not indifferent to the payoffs of their own group and that a sizable proportion was also sensitive to the earnings of the victimized group. Similarly, the modal motive for contributing to B was to “harm the other group while benefiting my own group” (12 out of 33) while others wanted “to harm the other group” (5 out of 33). Hence, more than half of all subjects who contributed to project B claimed to do so in order to reduce the payoffs of others. Were participants who chose option C merely confused about this option? Results show that most participants who contributed to C did so “to harm the other group” (11 out of 18). When we study detailed information on who chose C, we find that of the participants who choose C, most chose it two times or more. Most participants who chose option C apparently did not do so by mistake or out of confusion. Clearly, the motivational evidence indicates that a sizable proportion of participants who contribute to projects B and C were fully aware of the negative consequences for the out-group, and claimed to do so specifically because of those negative consequences. Both their motives and behavior are consistent with “strong aggression.”

**Table 2:** **Motives to invest in available projects of participants in the agression condition**.

**Motive**	**Participants**
**PROJECT A**					
To increase earnings of my group	15
To increase earnings of my group w/o harming other group	13
It is the safest or most profitable project	11
Not invested	4
Unclear motive	3
Others in my group had invested	2
*All*	*48*
**PROJECT B**					
Not invested	15
To harm the other group while benefiting my group	12
It is the safest or most profitable project	6
To harm the other group	5
Unclear motive	5
Indifference with project A	3
Was testing	1
Others in my group had invested	1
*All*	*48*
**PROJECT C**					
Not invested	30
To harm the other group	11
Was testing	3
Unclear motive	2
To harm the other group w/o benefiting my group	1
Others in my group had invested	1
*All*	*48*

Notice that contributions to project B might also be higher than contributions to C because B is a more effective aggression technology than C: Reducing the out-group's payoff by one MU costs 1 MU with C but only 1/3 of a MU with B. While we cannot rule out that some subjects chose B simply because it is a better punishment technology, we exploit again the survey on motivational aspects to show that this feature of our design is unlikely to drive the main results. As displayed in Table 2 only a small share of participants (5 out of 33) use B because it can “harm the other group.” By contrast a sizable proportion of subjects (12 out of 33) who contributed to B claimed to do so because it entailed a benefit for their “in group” and a cost to the “out-group,” consistent with an interpretation of parochial behavior.

**Figure 2:**
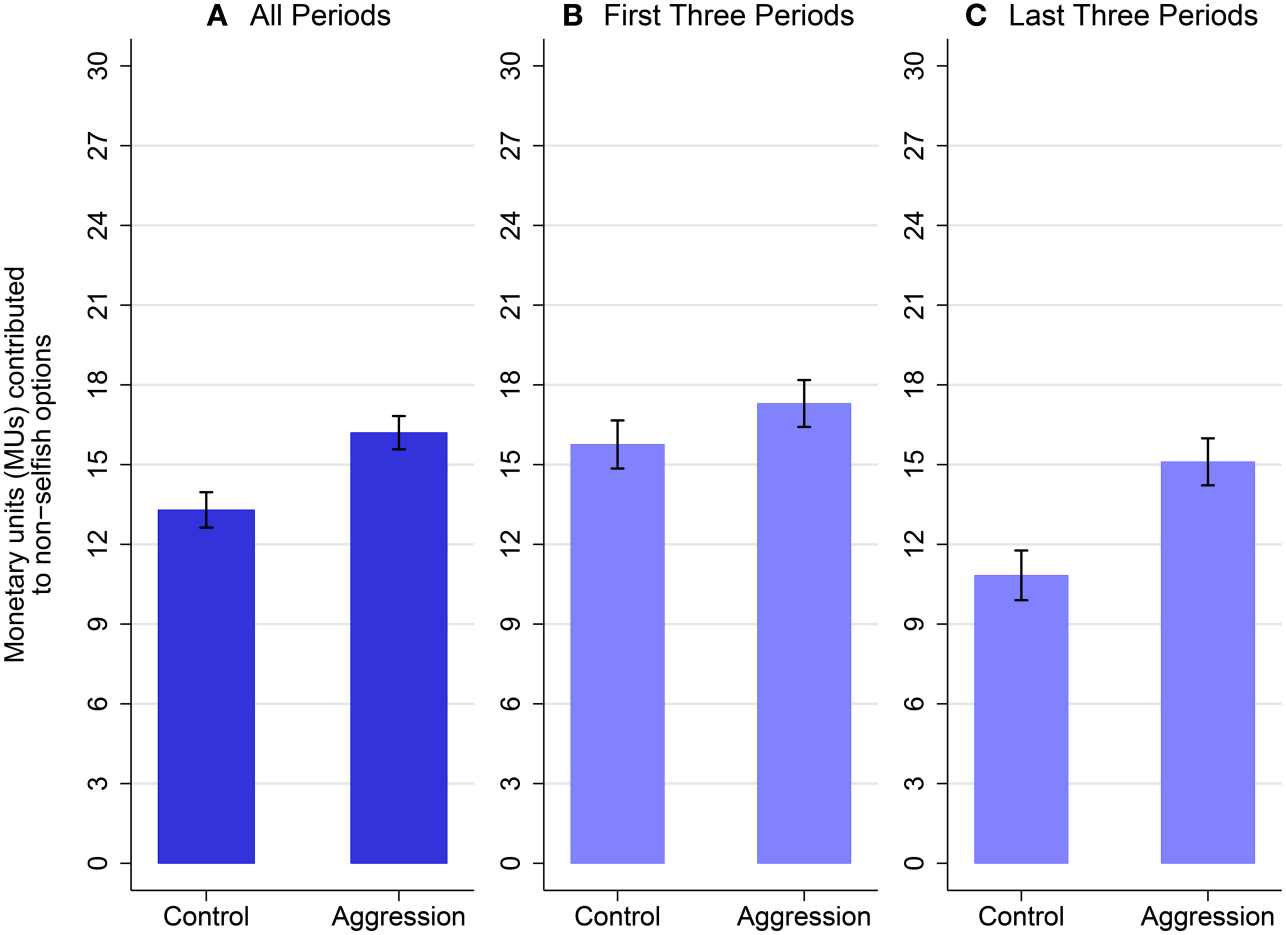
**This figure shows the number of monetary units (MUs) contributed to non-selfish projects (mean ± 1 standard error)**. Contributions to non-selfish projects are contributions to project A for individuals in the control condition, and contributions to projects A, B, and C for individuals in the aggression condition. The figure shows contributions over all six periods in **(A)**, over the first three out of six periods in **(B)**, and contributions over the last three out of six periods in **(C)**. Over all periods, the possibility to perpetrate aggression increases non-selfish behavior (*t* = 1.91, 0.05 < *p* < 0.1, c.f., Table 3 col. 2). The possibility to perpetrate aggression does not increase non-selfish behavior in the early periods (*t* = 0.86, *p* > 0.10, c.f., Table S1 col. 3), but it does so in the last three periods (*t* = 2.60, 0.01 < *p* < 0.05, c.f., Table S1 col. 4).

The second result is that non-selfish contributions increase if out-group aggression can be exerted. Figure 2 shows that contributions to non-selfish options (i.e., projects A, B, and C) are larger in the aggression condition than in the control condition (*t* = 1.91, *p* = 0.065, see Table 3, col. 2). The aggressive options partially crowd out project A. But in net, there is a 21% increase in non-selfish contributions. Importantly, the difference is even more pronounced at later periods of the experiment, as column (4) of Table 3 shows. The period-trend of contributions to non-selfish options is significantly bigger in the aggression condition than in the control condition (*t* = 2.73, *p* = 0.01). Repeated interactions create a strategic motive to contribute to the public good in early rounds of the game but this motive is less important in the last periods of the game. Indeed, there is a strong decrease in contributions to the public good in the control condition (see Section 1 and Table S1 in the Supplementary Material for additional support). This breakdown of in-group cooperation in the control condition is a well-known dynamic pattern in repeated public good games (Isaac and Walker, 1988; Ledyard, 1995; Fehr and Fischbacher, 2003). We interpret this dynamic pattern as a support to the evolutionary theories of parochial altruism (i.e., co-evolution of in-group cooperation and out-group aggression).

**Table 3:** **Aggression and non-selfish behavior**.

**Dependent variable: contributions to projects A, B, and C OLS estimates**			
	**(1)**	**(2)**	**(3)**
Aggressor	2.906	2.906	−2.004
	(1.878)	(1.520)^*^	(2.339)
Aggressor × Period			1.403
			(0.514)^**^
Period			−1.885
			(0.420)^***^
Constant	13.295	13.580	18.103
	(1.378)^***^	(2.801)^***^	(3.146)^***^
Observations	576	576	576
*R*^2^	0.017	0.136	0.143
Session dummies	No	Yes	Yes
Period dummies	No	Yes	No

Standard errors clustered by group in parentheses.

*** p < 0.01,

** p < 0.05,

* *p < 0.1*.

*Aggressor = 1 if participant in Aggression condition, = 0 if Control condition*.

Can unpacking effects due to the larger number of options in the aggression condition explain our results? Participants who play a public goods game with two identical public goods options tend to contribute much more in early rounds, but they learn over time that unpacked options are identical and their contributions converge to the level observed in the standard game in later rounds (Bernasconi et al., 2009). Our results are different. Contributions to projects A, B, and C in the aggression condition are similar to contributions to project A in the control condition in initial rounds. The difference in contributions becomes only salient toward later periods of the game. Thus, the pattern of our empirical findings is not consistent with unpacking effects. What is more, we carefully explained to participants that option A had no implications for other participants whereas option B did, and participants answered a series of control questions that specifically addressed this issue before making decisions (c.f. instructions in Section 2 of the Supplementary Material).

**Figure 3:**
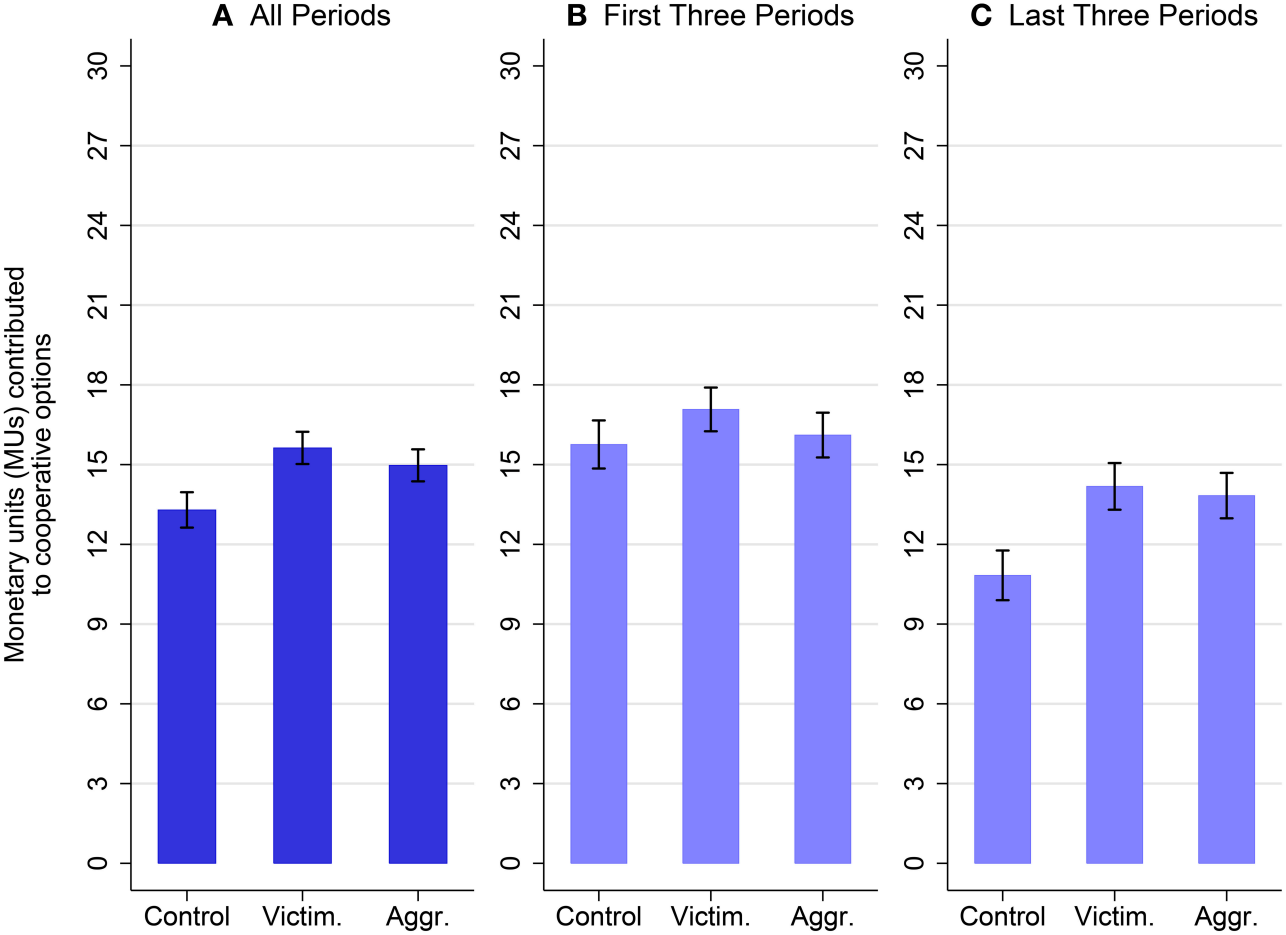
**This figure shows the number of monetary units (MUs) contributed to cooperative projects, i.e., projects that help the in-group (mean ± 1 standard error)**. Cooperative contributions are contributions to project A for individuals in the control and victimization conditions, and contributions to projects A and B for individuals in the aggression condition. The figure shows contributions over all six periods in **(A)**, over the first three out of six periods in **(B)**, and contributions over the last three out of six periods in **(C)**. Over all periods, the possibility to perpetrate aggression does not increase cooperative behavior. However, the possibility to perpetrate aggression does increase cooperative behavior in the last three periods (*t* = 1.70, 0.05 < *p* < 0.1, c.f., Table S2 col. 4). Moreover, in-group cooperation of victimized participants is also larger than cooperation of control participants in the last three periods (*t* = 2.01, 0.05 < *p* < 0.1, c.f., Table S3 col. 5).

Results are similar when we focus more specifically on in-group cooperation (i.e., projects A and B only). Figure 3 shows a positive impact of the possibility to perpetrate aggression on in-group cooperation, though the effect is not statistically significant over all periods (see Table 4, col. 2). The difference in cooperation between aggression and control is, however, magnified in the later periods of the game as the period-trend is significantly higher in the aggression condition than in the control (*t* = 2.56, *p* = 0.015, see Table 4, col. 3. C.f. Table S2 in the Supplementary Material). This result reflects the fact that in-group cooperation breaks down in the control condition whereas it remains high in the aggression condition.

**Table 4:** **Aggression and cooperative behavior**.

**Dependent variable: contributions to projects A and B OLS estimates**			
	**(1)**	**(2)**	**(3)**
Aggressor	1.677	1.677	−3.158
	(1.780)	(1.509)	(2.375)
Aggressor × Period			1.382
			(0.539)^**^
Period			−1.885
			(0.420)^***^
Constant	13.295	13.476	17.884
	(1.378)^***^	(2.806)^***^	(3.167)^***^
Observations	576	576	576
*R*^2^	0.006	0.110	0.116
Session dummies	No	Yes	Yes
Period dummies	No	Yes	No

Standard errors clustered by group in parentheses.

*** p < 0.01,

** p < 0.05,

*p < 0.1. Aggressor = 1 if participant in Aggression condition, = 0 if Control condition.

Our third result concerns in-group cooperation among victimized groups. Figure 3 also discusses how exposure to aggression affects in-group cooperation among victimized groups. We observe an increase in cooperation among victimized individuals, i.e., members of groups experiencing aggression from others, compared to the control condition. The increase takes place already in the early periods and the period-trend is significantly higher among victims than among control participants (*t* = 2.06, p = 0.045, see Table 5 col. 4. C.f. Table S3 in the Supplementary Material), suggesting that in-group cooperation does not decrease over time among victims as it does among individuals in the control condition. Interestingly, cooperation is neither sensitive to the extent of aggression experienced in the previous round nor to the amount of aggression victims expect for the current round (see Table 5 col. 3). This result suggests that the mere threat of aggression triggers a higher level of in-group cooperation among victims.

**Table 5:** **Victimization and cooperative behavior**.

**Dependent variable: contributions to project A OLS estimates**				
	**(1)**	**(2)**	**(3)**	**(4)**
Victim	2.333	2.817	5.178	−1.562
	(1.781)	(1.712)	(2.142)^**^	(2.703)
Victim × Period				1.113
				(0.539)^**^
Period				−1.885
				(0.420)^***^
Aggression _*t*−1_			−0.096	
			(0.063)	
E_*t*_(aggression _*t*_)			−0.033	
			(0.105)	
Constant	13.295	12.910	12.937	17.459
	(1.378)^***^	(3.772)^***^	(3.556)^***^	(3.643)^***^
Observations	576	480	480	576
*R*^2^	0.012	0.106	0.119	0.096
Session dummies	No	Yes	Yes	Yes
Period dummies	No	Yes	Yes	No
Sample restriction	No	period> 1	period> 1	No

Standard errors clustered by group in parentheses.

*** p < 0.01,

** p < 0.05,

*p < 0.1. Victim = 1 participant in Victimization condition, = 0 if Control condition; Aggression = total contribution of aggressors to projects B and C if participant in Victimization condition, = 0 if participant in Control condition.

## 4. Discussion

This study provides behavioral evidence consistent with the phenotypic predictions of evolutionary theories of parochial altruism.

Firstly, a proportion of participants in our experiment exhibit *strong aggression* in the absence of any strategic motive. Despite the fact that any level of desired in-group cooperation is attainable without perpetrating aggression toward an out-group, and that aggression does not entail any gains to the individual nor to her in-group over and above what can be attained without aggression, some participants do invest part of their endowment in reducing the payoffs of victimized groups. That is, participants in the aggression condition contribute a statistically significant amount of their endowment to anti-social options (projects B and, to a lesser extent, C) and are aware of the consequences for the other group. Aggression is not observed in isolation but *combined with in-group cooperation*. Participants readily perpetrate aggression toward a victimized group when this behavior favors the in-group, even if the strategic incentives for cooperation are also weak. Second, evolutionary theories of parochial altruism state that the presence of a type of behavior that combines in-group cooperation with out-group aggression is a sufficient condition for evolutionary stability of in-group altruism (Choi and Bowles, 2007). Consistent with this hypothesis, we find *more cooperative behavior that remains stable across periods* when participants have the possibility to perpetrate aggression against a victimized group, whereas there is a distinct drop in public-good contributions in the control condition, possibly as repeated-game/reputation mechanisms fade in that condition (Fischbacher and Gachter, 2010). This suggests that the possibility to exert aggression against an out-group is a more powerful mechanism to generate cooperation than the strategic concern to appear pro-social in the standard public goods setting. Third, we also document that the mere threat of aggression triggers a higher level of in-group cooperation among victims.

The asymmetric design of our experiment, in which groups that experience aggression cannot retaliate is key to identify the effects across victims and perpetrators. In particular, our results show that both for victims and perpetrators, exposure to aggression at the *group-level* increases in-group cooperation and indicate that the mere presence of aggression, much more than its extent, triggers higher cooperation. These results contrast with previous evidence on the deleterious pro-social impact of exposure to aggression when it occurs *at the individual level* (Alesina and La Ferrara, 2002). This moderating effect of group salience makes clear that the social context in which aggression takes place is a key factor for understanding the differential evolutions of large-scale cooperation observed in various post-conflict and war episodes (Bellows and Miguel, 2009; Blattman and Miguel, 2010). For example, micro-level studies interested in the reintegration of child soldiers find contrasted effects of exposure to civil war on political participation and local collective action (Humphreys and Weinstein, 2007; Blattman, 2009; Annan and Blattman, 2010).

Various organizations and institutions try to exploit the behavioral pattern of parochial altruism by creating a strong sense of community (as do various human-resource policies or even military training) and, at the same time, by making salient the looming threat of competition from other organizations. Our results suggest that such policies are doubly effective and that the creation of an outside threat is an effective stabilizer of in-group cooperation. We believe that this dual phenomenon is at the root of many genocides and large-scale atrocities that are perpetrated in the sake of in-group interests—e.g., the infamous “Radio-Television libre des Mille Collines” that exacerbated the Hutu-Tutsi ethnic divide and allowed Hutus to feel justified in committing murders during the Rwandan genocide.

In our experimental protocol, we neither manipulate the salience of group identity nor the emotional state of the participants. We believe that both would be promising avenues for future research. Indeed we expect that both an increase in group salience or getting subjects into an aggressive emotional state could amplify the complementarity between in-group pro-sociality and out-group anti-sociality; we present a lower bound to this complementarity.

### Conflict of interest statement

The authors declare that the research was conducted in the absence of any commercial or financial relationships that could be construed as a potential conflict of interest.

## Footnotes

^1^This is important because (Brewer, 2001) argues that “the relationship between in-group identification and out-group hostility is progressive and contingent rather than necessary or inevitable.” Indeed, Duckitt and Mphuthing (1998) show that out-group hostility is contingent on historical experience in the South African context.

## References

[B1] Abbink K. Brandts J. Herrmann B. Orzen H. (2010). Intergroup conflict and intra-group punishment in an experimental contest game. Am. Econ. Rev. 100, 420–447. 10.1257/aer.100.1.420

[B2] Alesina A. La Ferrara E. (2002). Who trusts others? J. Public Econ. 85, 207–234. 10.1016/S0047-2727(01)00084-6

[B3] Alexander M. Fotini C. (2011). Context modularity of human altruism. Science 334, 1392–1394. 10.1126/science.1202599 22158815

[B4] Annan J. Blattman C. (2010). The consequences of child soldiering. Rev. Econ. Stat. 92, 882–898. 10.1162/REST_a_0003621831429

[B5] Avilés L. (2002). Solving the freeloaders paradox: genetic associations and frequency-dependent selection in the evolution of cooperation among nonrelatives. Proc. Natl. Acad. Sci. U.S.A. 99, 14268-14273. 10.1073/pnas.212408299 12381790 PMC137873

[B6] Baron J. (2001). Confusion of group-interest and self-interest in parochial cooperation on behalf of a group. J. Confl. Resolut. 45, 283–296. 10.1177/0022002701045003002

[B7] Bell A. V. Richerson P. J. McElreath R. (2009). Culture rather than genes provides greater scope for the evolution of large-scale human prosociality. Proc. Natl. Acad. Sci. U.S.A. 106, 17671–17674. 10.1073/pnas.0903232106 19822753 PMC2764900

[B8] Bellows J. Miguel E. (2009). War and collective action in sierra leone. J. Public Econ. 93, 1144–1157. 10.1016/j.jpubeco.2009.07.012

[B9] Bernasconi M. Corazzini L. Kube S. Maréchal M. A. (2009). Two are better than one! individuals' contributions to “unpacked” public goods. Econ. Lett. 104, 31–33. 10.1016/j.econlet.2009.03.015

[B10] Blattman C. Miguel E. (2010). Civil war. J. Econ. Lit. 48, 3–57. 10.1257/jel.48.1.3

[B11] Blattman C. (2009). From violence to voting: war and political participation in uganda. Am. Polit. Sci. Rev. 103, 231–247. 10.1017/S0003055409090212

[B12] Bornstein G. Ben-Yossef M. (1994). Cooperation in intergroup and single-group social dilemmas. J. Exp. Soc. Psychol. 30, 52–67.

[B13] Bowles S. (2006). Group competition, reproductive leveling, and the evolution of human altruism. Science 314, 1569–1572. 10.1126/science.1134829 17158320

[B14] Bowles S. (2009). Did warfare among ancestral hunter-gatherers affect the evolution of human social behaviors? Science 324, 1293–1298. 10.1126/science.1168112 19498163

[B15] Boyd R. Richerson P. J. (2006). Roots of Human Sociality Chapter Culture and the Evolution of the Human Social Instincts. Oxford: Berg.

[B16] Boyd R. Gintis H. Bowles S. Richerson P. J. (2003). The evolution of altruistic punishment. Proc. Natl. Acad. Sci. U.S.A. 100, 3531–3535. 10.1073/pnas.0630443100 12631700 PMC152327

[B17] Brewer M. (2001). Social Identity, Intergroup Conflict, and Conflict Reduction Chapter 2 Ingroup Identification and Intergroup Conflict: When Does In-Group Love Become Out-Group Hate? Oxford, UK: Oxford University Press.

[B18] Chagnon N. A. (1988). Life histories, blood revenge, and warfare in a tribal population. Science 239, 985–992. 17815700 10.1126/science.239.4843.985

[B19] Choi J.-K. Bowles S. (2007). The coevolution of parochial altruism and war. Science 318, 363–340. 10.1126/science.1144237 17962562

[B20] Darwin C. (1871). The Descent of Man and Selection in Relation to Sex, 2nd Edn. New York, NY: American Home Library.

[B21] De Dreu C. K. W. Greer L. L. Handgraaf M. J. J. Shalvi S. Van Kleef G. A. Baas M.. (2010). The neuropeptide oxytocin regulates parochial altruism in intergroup conflict among humans. Science 328, 408–411. 10.1126/science.1189047 20538951

[B22] Doyle M. Sambanis N. (2006). Making War and Building Peace: United Nations Peace Operations. Princeton, NJ: Princeton University Press.

[B23] Duckitt J. Mphuthing T. (1998). Group identification and intergroup attitudes: a longitudinal analysis in south africa. J. Pers. Soc. Psychol. 74, 80–85. 9457777 10.1037//0022-3514.74.1.80

[B24] Efferson C. Lalive R. Fehr E. (2008). The coevolution of cultural groups and ingroup favoritism. Science 321, 1844–1849. 10.1126/science.1155805 18818361

[B25] Fehr E. Fischbacher U. (2003). The Nature of human altruism. Nature 425, 785–791. 10.1038/nature02043 14574401

[B26] Fischbacher U. Gachter S. (2010). Social preferences, beliefs, and the dynamics of free riding in public goods experiments. Am. Econ. Rev. 100, 541–556. 10.1257/aer.100.1.541

[B27] Fischbacher U. (2007). z-Tree: Zurich toolbox for ready-made economic experiments. Exp. Econ. 10, 171–178. 10.1007/s10683-006-9159-4

[B28] Franck S. (2003). Repression of competition and the evolution of cooperation. Evolution 57, 693–705. 10.1111/j.0014-3820.2003.tb00283.x 12778541

[B29] Gat A. (2006). War in Human Civilization. Oxford, UK: Oxford University Press.

[B30] Goette L. Huffman D. Meier S. (2006). The impact of group membership on cooperation and norm enforcement: evidence using random assignment to real social groups. Am. Econ. Rev. 96, 212–216. 10.1257/000282806777211658

[B31] Goette L. Huffman D. Meier S. Sutter M. (2012). Competition between organizational groups: its impact on altruistic and antisocial motivations. Manage. Sci. 58, 948–960. 10.1287/mnsc.1110.1466

[B32] Halevy N. Bornstein G. Sagiv L. (2008). “in-group love” and “out-group hate” as motives for individual participation in intergroup conflict: a new game paradigm. Psychol. Sci. 19, 405–411. 10.1111/j.1467-9280.2008.02100.x 18399895

[B33] Hamilton W. (1975). Biosocial Anthropology Chapter Innate Social Aptitudes of Man: An Approach from Evolutionary Genetics. New York, NY: John Wiley & Sons.

[B34] Henrich J. McElreath R. Barr A. Ensminger J. Barrett C. Bolyanatz A.. (2006). Costly punishment across human societies. Science 312, 1767–1770. 10.1126/science.1127333 16794075

[B35] Henrich J. Ensminger J. McElreath R. Barr A. Barrett C. Bolyanatz A.. (2010). Markets, religion, community size, and the evolution of fairness and punishment. Science 327, 1480. 10.1126/science.1182238 20299588

[B36] Humphreys M. Weinstein J. (2007). Demobilization and reintegration. J. Conflict Resolut. 51, 531–567. 10.1177/0022002707302790

[B37] Isaac R. Walker J. (1988). Group size effects in public goods provision: the voluntary contributions mechanism. Q. J. Econ. 103, 179–199.

[B38] Keeley L. (1996). War Before Civilization. New York, NY: Oxford University Press.

[B39] Ledyard J. (1995). Handbook of Experimental Economics Chapter Public Goods: a Survey of Experimental Research. Princeton, NJ: Princeton University Press.

[B40] Lehmann L. Feldman M. (2008). War and the evolution of belligerence and bravery. Proc. R. Soc. Lond. B Biol. Sci. 275, 2877–2885. 10.1098/rspb.2008.0842 18755675 PMC2605837

[B41] Lehmann L. (2011). The demographic benefits of belligerence and bravery: defeated group repopulation or victorious group size expansion? PLoS ONE 6:e21437. 10.1371/journal.pone.0021437 21750712 PMC3130041

[B42] Mathew S. Boyd R. (2011). Punishment sustains large-scale cooperation in prestate warfare. Proc. Natl. Acad. Sci. U.S.A. 108, 11375-11380. 10.1073/pnas.1105604108 21670285 PMC3136302

[B43] Posner D. (2004). The political salience of cultural difference: why chewas and tumbukas are allies in zambia and adversaries in malawi. Am. Polit. Sci. Rev. 98, 529–545. 10.1017/S0003055404041334

[B44] Sambanis N. Schulhofer-Wohl J. Shayo M. (2012). Parochialism as a central challenge in counterinsurgency. Science 336, 805–808. 10.1126/science.1222304 22605736

[B45] Tan J. H. Bolle F. (2007). Team competition and the public goods game. Econ. Lett. 96, 133–139. 10.1016/j.econlet.2006.12.031

[B46] von Goethe J. W. (1808). Faust. Der Tragödie Erster Teil, (Berlin).

[B47] Wilson E. O. (1975). Sociobiology: The New Synthesis. Cambridge, MA: Harvard University Press.

[B48] Yamagishi T. Mifune N. (2009). Social exchange and solidarity: in-group love or out-group hate? Evol. Hum. Behav. 30, 229–237. 10.1016/j.evolhumbehav.2009.02.00418399895

[B49] Zizzo D. J. (2004). Inequality and procedural fairness in a money-burning and stealing experiment, in Inequality, Welfare and Income Distribution: Experimental Approaches (Research on Economic Inequality, Volume 11), ed Cowell F. (Elsevier: Emerald Group Publishing Limited), 215–247.

