# Supplementary material for: Do we harm others even if we don't need to?
Source: Front Psychol. 2015 Jun 2;6:729. doi: 10.3389/fpsyg.2015.00729 (PMC4451738; doi:10.3389/fpsyg.2015.00729)
Supplement: Supplementary file 1 [file Presentation1.PDF]

Supplementary Material: Do We Harm Others Even if We Don't Need To?

Maria Paula Cacault [1], Lorenz Goette [1], Rafael Lalive [1,*] and Mathias Thoenig [1]

[1] *Department of Economics, University of Lausanne, Switzerland*

Correspondence*:
Rafael Lalive
University of Lausanne, Quartier UNIL-Dorigny, Batiment Internef, 1015 Lausanne, Switzerland, rlalive@unil.ch

Parochial Altruism: Pitfalls and Prospects

1 THE STATISTICAL MODEL

In order to explain non-selfish and cooperative behaviors of participants in the different conditions, we estimate the following general statistical model

$$Y_{i,t} = x'_{i,t}\beta + \gamma D_i + \varepsilon_{i,t} \tag{1}$$

where $Y_{i,t}$ is total non-selfish or cooperative contributions of individual i in period $t = 1, \ldots, 6$; D_i is a condition binary (or dummy) variable and $X_{i,t}$ are controls that may include session and period dummies and actual or expected aggression received (in the Victimization condition).

The residuals of this model $\varepsilon_{i,t}$ cannot be treated as independent, because the decisions of participants within a group may be correlates. Moreover, residuals of an individual i are probably correlated in time. Inference from this model should thus be made using cluster-robust standard errors. With G groups and N individuals, the covariance matrix that allows for clustering (as well as heteroskedasticity) is given by

$$\Sigma_C = (X'X)^{-1}\Big(\sum_g X'_g \hat{\Psi}_g X_g\Big)(X'X)^{-1} \tag{2}$$

where $X = [x' \quad D]$, X_g the matrix of regressors of group g, and $\hat{\Psi}_g = q\hat{e}_g\hat{e}'_g$, with $q = G(N-1)[(N-k)(G-1)]^{-1}$ a degrees-of-freedom adjustment (**Angrist and Pischke**, 2009). Note that the clustered estimator of the covariance matrix is consistent when the number of groups is large. In our setup, we have 32 matching groups which is arguably not "large" enough. Unreported bootstrapped standard errors of the treatment variables are between 0 and 10% larger than the cluster-robust standard errors presented in the results, depending on the specification. This suggests that 32 clusters are enough in our case to rely on the asymptotics needed for valid inference from clustered-robust standard errors.

An alternative approach to gain precision is to exploit the knowledge of the correlation structure given by the experimental design. In particular, there are some restrictions we can impose on the elements of the matrix of the cross-product of residuals $\hat{e}_g\hat{e}'_g$. Individual decisions are simultaneous, so one can safely assume that the decision of individual i at time t is uncorrelated to the decisions of other in-group members at time t, and set the corresponding elements of $\hat{e}_g\hat{e}'_g$ to zero. Moreover, at the end of each period, only the information about the decisions of in-group members in the previous period is displayed. Hence, one

could assume that the decision of individual i at time t is uncorrelated to the decisions of other in-group members at time $t \pm k$, with $k > 1$. In other words, one can consider standard errors that are clustered by individual, but that allow for first-order correlation between the decisions of individuals within a group.

Imposing these restrictions on the covariance matrix, we get pseudo-clustered or generalized standard errors (GSE). We assume that the test statistics constructed with these standard errors follow the same distribution as the test statistics constructed with the standard errors in (2), that are clustered by group (CSE), and consider the critical values from a t-distribution with $G-1$ degrees of freedom. As a robustness check, we also contrasted the t-statistics against the distribution of test statistics constructed with standard errors that are clustered by individual. The significance of estimates remained the same.

Both sets of standard errors are reported in tables S1, S2, and S3. These tables complement the results presented in tables 3, 4 and 5 in the main text.

2 INSTRUCTIONS USED IN THE EXPERIMENT

(The following instructions were originally written in French.)

Instructions for this experiment.

Welcome to this experiment! You will have to make decisions that will affect your earnings as well as the earnings of other participants. Although we express all earnings in terms of points, these points will be exchanged at the end of the experiment using the following exchange rate:

20 points = CHF 1.-

From this moment, it is **strictly forbidden to talk with other participants**. If you have any questions, please contact the assistants. If you do not follow this rule, we will have to exclude you from the experiment.

(Control: the following part was contained in the instructions distributed to Control participants)

There are six participants in the room wearing an ORANGE shirt. Your group is composed of three members: you and two other participants wearing an ORANGE shirt. Hence, there are two different groups wearing orange shirts, but you will not know which participants are the two other members of your group.

a) What is it about?

You will confront the following situation in six consecutive rounds. In each round, you will receive 30 points. You can allocate them freely, entirely or partially, in the following project:

Project: Every point invested in the project costs you one point, but gives two points to your group (the point you invested plus an additional point). These two points will then be distributed evenly among the three members of your group, including yourself.

For example, if you invest 3 points in the project, it costs you 3 points but gives 6 points to your group. From these 6 points, every member of your group, including yourself, receives 2 points. Hence, the two

other members of your group have won 2 points each and that cost you 1 point net (you first invested 3 points and then got 2 points).

You are free not to invest all your points by keeping them to yourself.

To sum up, here is how your earnings are computed in each round:

Your earnings = 30 points - the points that you invested in the project + your part of the project, that is: 1/3 of (2 * the total number of points that your group invested in the project)

The following examples show you again how the earnings of each participant are computed in each round:

Example 1: you don't invest any point in the project. The other members of your group do not invest any point in the project either. Your earnings (equal to the earnings of the other members of your group) are:

Your earnings = 30 points - 0 point that you invested in the project + your part of the project, that is: 1/3 of (2 * 0 point that your group invested in the project) = 30 points

Example 2: you invest 30 points in the project. The other members of your group invest 30 points each in the project. Your earnings (equal to the earnings of the other members of your group) are:

Your earnings = 30 points - 30 points that you invested in the project + your part of the project, that is: 1/3 of (2 * 90 points that your group invested in the project) = 60 points

Example 3: you don't invest any point in the project. The other members of your group invest 30 points each in the project. Your earnings and the earnings of the other members of your group are:

Your earnings = 30 points - 0 point that you invested in the project + your part of the project, that is: 1/3 of (2 * 60 points that your group invested in the project) = 70 points

Earnings of each of the other two members of your group = 30 points - 30 points that she/he invested in the project + her/his part of the project, that is: 1/3 of (2 * 60 points that your group invested in the project) = 40 points

b) What will you do?

In each round, you have to decide how many points you want to invest in the project. If you want to invest or keep 0 point, you have to type ”0” in the corresponding cell. You will do this using the following screen: (Fig. S1)

Afterwards, you will give your estimation of what the other members of your group have done. This estimation will not have any consequence on your earnings or on the earnings of other participants. You will do this using the following screen: (Fig. S2)

At the end of each round, your earnings will be shown as well as other information regarding the decisions of the other members of your group. They will be presented in the following screen: (Fig. S3)

c) Did you understand?

Before the six rounds that will count for your earnings in this experiment start, we want to be sure that you, and all other participants, have understood the decisions to be made. For this, please answer the following questions. When you have finished, raise your hand so that assistants come check your answers.

Question 1: you don't invest any point in the project. The other members of your group invest 30 points each in the project. What are your earnings? What are the earnings of the other members of your group?

Question 2: you invest 5 points in the project. The two other members of your group invest 20 points each in the project. What are your earnings? What are the earnings of the other members of your group?

Question 3: you invest 10 points in the project. The two other members of your group also invest 10 points each in the project. What are your earnings? What are the earnings of the other members of your group?

(Aggression: the following part was contained in the instructions distributed to Aggressors)

There are six participants in the room wearing a GREEN shirt. Your group is composed of three members: you and two other participants wearing a GREEN shirt. Hence, there are two different groups wearing green shirts, but you will not know which participants are the two other members of your group.

Participants wearing a BLUE shirt are also assigned to two groups of three participants each. One of these groups is connected to yours. We will explain in what follows what this means. However, members of the blue group will be confronted to a different situation than yours and their decisions during this experiment will not have any impact whatsoever on your earnings.

a) What is it about?

You will confront the following situation in six consecutive rounds. In each round, you will receive 30 points. You can allocate them freely, entirely or partially, in the following projects:

Project A: Every point invested in project A costs you one point, but gives two points to your group (the point you invested plus an additional point). These two points will then be distributed evenly among the three members of your group, including yourself.

For example, if you invest 3 points in project A, it costs you 3 points but gives 6 points to your group. From these 6 points, every member of your group, including yourself, receives 2 points. Hence, the two other members of your group have won 2 points each and that cost you 1 point net (you first invested 3 points and then got 2 points).

Project B: Every point invested in project B costs you one point and, just like project A, gives two points to your group. The additional point is deducted from the income of the BLUE group that is connected to yours.

Let's consider the previous example. If you invest 3 points in project B, the earnings of your group is the same as before: the two other members of your group have won 2 points each and that cost you 1 point net. Moreover, the earnings of the BLUE group that is connected to yours is reduced by 3 points; that means that the earnings of each member of the BLUE group is reduced by 1 point.

Project C: Every point invested in project C costs you one point and it reduces the earnings of the BLUE group that is connected to yours by one point. This project does not change the earnings of your group.

For example, if you invest 3 points in project C, that cost you 3 points. The earnings of the two other members of your group do not change and you do not get points back. The earnings of the BLUE group that is connected to yours is reduced by 3 points; that means that the earnings of each member of the BLUE group is reduced by 1 point.

You can obviously allocate your points in more than one project. That is, you can invest some points in project A, some points in project B and some points in project C, if you want. You are also free not to invest all your points by keeping them to yourself. To sum up, here is how your earnings are computed in each round:

Your earnings = 30 points - the points that you invested in projects A, B and C + your part of projects A and B, that is: 1/3 of (2 * the total number of points that your group invested in projects A and B)

The following examples show you again how the earnings of each participant are computed in each round:

Example 1: you don't invest any point in the projects. The other members of your group do not invest any point in the projects either. Your earnings (equal to the earnings of the other members of your group) are:

Your earnings = 30 points - 0 point that you invested in projects A, B and C + your part of projects A and B, that is: 1/3 of (2 * 0 point that your group invested in projects A and B) = 30 points

Given that your group did not invest in projects B and C, there is no reduction in the earnings of the members of BLUE group that is connected to yours.

Example 2: you invest 30 points in project A. The other members of your group invest 30 points each in project A. Your earnings (equal to the earnings of the other members of your group) are:

Your earnings = 30 points - 30 points that you invested in projects A, B and C + your part of projects A and B, that is: 1/3 of (2 * 90 point that your group invested in projects A and B) = 60 points

Given that your group did not invest in projects B and C, there is no reduction in the earnings of the members of BLUE group that is connected to yours.

Example 3: you invest 30 points in project B. The other members of your group invest 30 points each in project B. Your earnings (equal to the earnings of the other members of your group) are:

Your earnings = 30 points - 30 points that you invested in projects A, B and C + your part of projects A and B, that is: 1/3 of (2 * 90 point that your group invested in projects A and B) = 60 points

Given that your group invested a total of 60 points in project B, the total earnings of the BLUE group that is connected to yours are reduced by 60 points. That means that the earnings of each member of the BLUE group are reduced by 20 points.

Example 4: you don't invest any point in the projects. The other members of your group invest 30 points each in project A. Your earnings and the earnings of the other members of your group are:

Your earnings = 30 points - 0 point that you invested in projects A, B and C + your part of projects A and B, that is: 1/3 of (2 * 60 points that your group invested in projects A and B) = 70 points

Earnings of each of the other two members of your group = 30 points - 30 points that she/he invested in projects A, B and C + her/his part of projects A and B, that is: 1/3 of (2 * 60 points that your group invested in projects A and B) = 40 points

Given that your group did not invest in projects B and C, there is no reduction in the earnings of the members of BLUE group that is connected to yours.

Example 5: you don't invest any point in the projects. The other members of your group invest 30 points each in project B. Your earnings and the earnings of the other members of your group are:

Your earnings = 30 points - 0 point that you invested in projects A, B and C + your part of projects A and B, that is: 1/3 of (2 * 60 points that your group invested in projects A and B) = 70 points

Earnings of each of the other two members of your group = 30 points - 30 points that she/he invested in projects A, B and C + her/his part of projects A and B, that is: 1/3 of (2 * 60 points that your group invested in projects A and B) = 40 points

Given that your group invested a total of 60 points in project B, the total earnings of the BLUE group that is connected to yours are reduced by 60 points. That means that the earnings of each member of the BLUE group are reduced by 20 points.

Example 6: you invest 30 points in project C. The other members of your group invest 30 points each in project C. Your earnings (equal to the earnings of the other members of your group) are:

Your earnings = 30 points - 30 points that you invested in projects A, B and C + your part of projects A and B, that is: 1/3 of (2 * 0 point that your group invested in projects A and B) = 0 point

Given that your group invested a total of 90 points in project C, the total earnings of the BLUE group that is connected to yours are reduced by 90 points. That means that the earnings of each member of the BLUE group are reduced by 30 points.

b) What will you do?

In each round, you have to decide how many points you want to invest in the projects. If you want to invest or keep 0 point, you have to type ”0” in the corresponding cell. You will do this using the following screen: (Fig. S4)

Afterwards, you will give your estimation of what the other members of your group have done. This estimation will not have any consequence on your earnings or on the earnings of other participants. You will do this using the following screen: (Fig. S5)

At the end of each round, your earnings will be shown as well as other information regarding the decisions of the other members of your group. They will be presented in the following screen: (Fig. S6)

c) Did you understand?

Before the six rounds that will count for your earnings in this experiment start, we want to be sure that you, and all other participants, have understood the decisions to be made. For this, please answer the following questions. When you have finished, raise your hand so that assistants come check your answers.

Question 1: you don't invest any point in the projects. The other members of your group do not invest any points in the projects either. What are your earnings? What are the earnings of the other members of your group? What is the reduction in the earnings of each member of the BLUE group?

Question 2: you invest 5 points in project B. The two other members of your group invest 20 points each in project B. What is your income? What are your earnings? What are the earnings of the other members of your group? What is the reduction in the earnings of each member of the BLUE group?

Question 3: you invest 10 points in project A and 10 points in project C. The two other members of your group also invest 10 points in project A and 10 points in project C. What are your earnings? What are the earnings of the other members of your group? What is the reduction in the earnings of each member of the BLUE group?

(Victimization: the following part was contained in the instructions distributed to Victims)

There are six participants in the room wearing a BLUE shirt. Your group is composed of three members: you and two other participants wearing a BLUE shirt. Hence, there are two different groups wearing blue shirts, but you will not know which participants are the two other members of your group.

Participants wearing a GREEN shirt are also assigned to two groups of three participants each. One of these groups is connected to yours. We will explain in what follows what this means.

a) What is it about?

You will confront the following situation in six consecutive rounds. In each round, you will receive 30 points. You can allocate them freely, entirely or partially, in the following project:

Project A: Every point invested in project A costs you one point, but gives two points to your group (the point you invested plus an additional point). These two points will then be distributed evenly among the three members of your group, including yourself.

For example, if you invest 3 points in project A, it costs you 3 points but gives 6 points to your group. From these 6 points, every member of your group, including yourself, receives 2 points. Hence, the two other members of your group have won 2 points each and that cost you 1 point net (you invested first 3 points and then got 2 points).

You are free not to invest all your points by keeping them to yourself.

The GREEN group connected to yours is confronted to a somewhat different situation. The members of the GREEN group can freely allocate their 30 points, totally or partially, in the following projects:

Project A: This project is the same as your project A.

Project B: Every point invested in project B costs them one point and, just like project A, gives two points to their group. The additional point is deducted from the earnings of your BLUE group.

For example, if a member of the GREEN group invests 3 points in project B, this gives the same income to her/his group as the investment in project A: the two other members of her/his group win 2 points each and that cost her/him 1 point net. Moreover, the earnings of your BLUE group are reduced by 3 points; that means that the earnings of each of the three members of your BLUE group, including yourself, are reduced by 1 point.

Project C: Every point invested in project C costs them one point and it reduces the earnings of your BLUE group by one point. This project does not change the earnings of their group.

For example, if a member of the GREEN group invests 3 points in project C; that cost her/him 3 points. The earnings of the two other members of her/his group do not change and she/he does not get points back. The earnings of your BLUE group are reduced by 3 points; that means that the earnings of each of the three members of your BLUE group, including yourself, are reduced by 1 point.

To sum up, here is how your earnings are computed in each round:

Your earnings = 30 points - the points that you invested in project A + your part of project A, that is: 1/3 of (2 * the total number of points that your group invested in project A) - your part of the losses due to the investments of the GREEN group, that is: 1/3 of (the total number of points that the GREEN group invested in projects B and C)

The following examples show you again how the earnings of each participant are computed in each round:

Example 1: you don't invest any point in project A. The other members of your group do not invest any point in project A either. The GREEN group does not invest any point in projects B and C. Your earnings (equal to the earnings of the other members of your group) are:

Your earnings = 30 points - 0 point that you invested in project A + your part of project A, that is: 1/3 of (2 * 0 point that your group invested in project A) - your part of the losses due to the investments of the GREEN group, that is: 1/3 of (0 point that the GREEN group invested in projects B and C) = 30 points.

Example 2: you invest 30 points in project A. The other members of your group also invest 30 points each in project A. The GREEN group does not invest any point in projects B and C. Your earnings (equal to the earnings of the other members of your group) are:

Your earnings = 30 points - 30 points that you invested in project A + your part of project A, that is: 1/3 of (2 * 90 points that your group invested in project A) - your part of the losses due to the investments of the GREEN group, that is: 1/3 of (0 point that the GREEN group invested in projects B and C) = 60 points.

Example 3: you don't invest any point in project A. The other members of your group invest 30 points each in project A. The GREEN group does not invest any point in projects B and C. Your earnings and the earnings of the other members of your group are:

Your earnings = 30 points - 0 point that you invested in project A + your part of project A, that is: 1/3 of (2 * 60 point that your group invested in project A) - your part of the losses due to the investments of the GREEN group, that is: 1/3 of (0 point that the GREEN group invested in projects B and C) = 70 points.

Earnings of each of the other two members of your group = 30 points - 30 points that she/he invested in project A + her/his part of project A, that is: 1/3 of (2 * 60 points that your group invested in project A) - your part of the losses due to the investments of the GREEN group, that is: 1/3 of (0 point that the GREEN group invested in projects B and C) = 40 points.

Example 4: you invest 30 points in project A. The other members of your group also invest 30 points each in project A. The GREEN group invests a total of 30 points in projects B and C. Your earnings (equal to the earnings of the other members of your group) are:

Your earnings = 30 points - 30 point that you invested in project A + your part of project A, that is: 1/3 of (2 * 90 points that your group invested in project A) - your part of the losses due to the investments of the GREEN group, that is: 1/3 of (30 points that the GREEN group invested in projects B and C) = 50 points.

b) What will you do?

In each round, you have to decide how many points you want to invest in project A. If you want to invest or keep 0 point, you have to type ”0” in the corresponding cell. You will do this using the following screen: (Fig. S7)

Afterwards, you will give your estimation of what the other participants have done. This estimation will not have any consequence on your earnings or on the earnings of other participants. You will do this using the following screen: (Fig. S8)

At the end of each round, your earnings will be shown as well as other information regarding the decisions of the other members of your group. They will be presented in the following screen: (Fig. S9)

c) Did you understand?

Before the six rounds that will count for your earnings in this experiment start, we want to be sure that you, and all other participants, have understood the decisions to be made. For this, please answer the following questions. When you have finished, raise your hand so that assistants come check your answers.

Question 1: you don’t invest any point in project A. The other members of your group do not invest any points in project A either. The GREEN group does not invest any points in projects B and C. What are your earnings? What are the earnings of the other members of your group?

Question 2: you invest 5 points in project A. The other members of your group invest 20 points each in project A. The GREEN group does not invest any points in projects B and C. What are your earnings? What are the earnings of the other members of your group?

Question 3: you invest 20 points in project A. The other members of your group also invest 20 points each in project A. The GREEN group invests a total of 30 points in projects B and C. What are your earnings? What are the earnings of the other members of your group?

3 CLASSIFICATION OF MOTIVES TO INVEST IN AVAILABLE PROJECTS

At the end of each session, participants completed an open-answer survey that contained questions on why they chose to contribute to the projects available to them. Some examples of participants' answers are translated in Table S4.

Two independent raters classified the answers of participants in the aggression condition into the set of motives that are tabulated in Table 2 in the main text. The inter-rater agreement about this classification, as measured by Cohen's Kappa statistic (**Cohen**, 1960), is presented in Table S5. Inter-rater agreement is moderate regarding motives to contribute to project A ($\kappa = 0.5888$, $p < 0.001$), and very good regarding motives to contribute to projects B ($\kappa = 0.8431$, $p < 0.001$) and C ($\kappa = 0.8866$, $p < 0.001$). Whenever there was a disagreement between the raters, the classification of a third independent rater was used to resolve rater disagreement. If the third rater's classification matched the first rater's classification, we kept the first rater's classification. If the third rater's classification matched the second rater's classification, we kept the second classification. In case of disagreement between the three raters, the answer was classified as "unclear motive".

REFERENCES

Angrist, J. D. and Pischke, J.-S. (2009), Mostly Harmless Econometrics. An Empiricist's Companion (Princeton University Press)

Cohen, J. (1960), A coefficient of agreement for nominal scales, *Educational and Psychological Measurement*, 20, 1, 37–46

4 SUPPLEMENTARY TABLES AND FIGURES

Table S1. Aggression and non-selfish behavior

Dependent variable: contributions to projects A, B and C<br>OLS estimates				
	(1)	(2)	(3)	(4)
Aggressor	2.906	2.906	1.542	4.271
	(1.878)	(1.520)*	(1.733)	(1.712)**
	[1.713]*	[1.497]*	[1.783]	[1.645]**
Constant	13.295	13.580	14.941	10.135
	(1.378)***	(2.801)***	(2.325)***	(3.727)**
	[1.229]***	[2.819]***	[2.873]***	[3.553]***
Observations	576	576	288	288
R^2	0.017	0.136	0.110	0.142
Sample restriction	No	No	period$\leq$3	period>3
Session dummies	No	Yes	Yes	Yes
Period dummies	No	Yes	Yes	Yes

Notes: Standard errors clustered by group (CSE) in parentheses. Generalized standard errors (GSE) in brackets (clustered by individual and allowing for correlation between individual i's choice in period t with the decisions of her in-group members in $t-1$).*** p<0.01, ** p<0.05, * p<0.1 (t-dist. with $G-1$ degrees of freedom, where G is the number of clusters. GSE assume same distribution as CSE).
Aggressor=1 if participant in Aggression condition, =0 if Control condition.

Table S2. Aggression and cooperative behavior

Dependent variable: contributions to projects A and B<br>OLS estimates				
	(1)	(2)	(3)	(4)
Aggressor	1.677	1.677	0.354	3.000
	(1.780)	(1.509)	(1.716)	(1.762)*
	[1.624]	[1.458]	[1.751]	[1.648]*
Constant	13.295	13.476	14.795	9.760
	(1.378)***	(2.806)***	(2.260)***	(3.789)**
	[1.229]***	[2.725]***	[2.779]***	[3.479]***
Observations	576	576	288	288
R^2	0.006	0.110	0.087	0.108
Sample restriction	No	No	period$\leq$3	period>3
Session dummies	No	Yes	Yes	Yes
Period dummies	No	Yes	Yes	Yes

Notes: Standard errors clustered by group (CSE) in parentheses. Generalized standard errors (GSE) in brackets (clustered by individual and allowing for correlation between individual i's choice in period t with the decisions of her in-group members in $t-1$).*** p<0.01, ** p<0.05, * p<0.1 (t-dist. with $G-1$ degrees of freedom, where G is the number of clusters. GSE assume same distribution as CSE).

Aggressor=1 if participant in Aggression condition, =0 if Control condition.

Table S3. Victimization and cooperative behavior

	Dependent variable: contributions to project A<br>OLS estimates				
	(1)	(2)	(3)	(4)	(5)
Victim	2.333	2.817	5.178	1.319	3.347
	(1.781)	(1.712)	(2.142)**	(1.997)	(1.662)*
	[1.601]	[1.405]*	[1.935]**	[1.872]	[1.551]**
Aggression_{t-1}			-0.096		
			(0.063)		
			[0.060]		
$\text{E}_t(\text{aggression}_t)$			-0.033		
			(0.105)		
			[0.096]		
Constant	13.295	12.910	12.937	12.333	12.017
	(1.378)***	(3.772)***	(3.556)***	(2.697)***	(4.160)***
	[1.229]***	[2.984]***	[2.850]***	[2.778]***	[3.603]***
Observations	576	480	480	288	288
R^2	0.012	0.106	0.119	0.075	0.096
Sample restriction	No	period>1	period>1	period≤3	period>3
Session dummies	No	Yes	Yes	Yes	Yes
Period dummies	No	Yes	Yes	Yes	Yes

Notes: Standard errors clustered by group (CSE) in parentheses. Generalized standard errors (GSE) in brackets (clustered by individual and allowing for correlation between individual i's choice in period t with the decisions of her in-group members in $t-1$).*** p<0.01, ** p<0.05, * p<0.1 (t-dist. with $G-1$ degrees of freedom, where G is the number of clusters. GSE assume same distribution as CSE).

Victim=1 participant in Victimization condition, =0 if Control condition; *Aggression* = total contribution of aggressors to projects B and C if participant in Victimization condition, =0 if participant in Control condition.

Table S4. Examples of participants' motives to invest in available projects

Motive	Examples of participants' statements
If you invested in project A, why did you do it?	
To increase earnings of my group:	"To gain the trust of other members of my group, so they would also invest and we could maximize global profits"<br>"Because one point invested yielded two points to my group"
To increase earnings of my group w/o hurting the other group:	"It was the one that yielded the best, and did not penalize the blue team, whose decisions did not influence our earnings: it's a sort of equality, of justice that I tried to give through my investments"<br>"Because it yields points without touching the earnings of the blue group and everybody is better off"
It is the safest or most profitable project:	"Because an investment in project A has little risk"
If you invested in project B, why did you do it?	
To harm the other group while benefiting my group:	"To increase by the same quantity (as project A) the points of my group but trying to penalize the blue group"<br>"To reduce earnings of the blue group and make my team earn points"
It is the safest or most profitable project:	"I think it was the most profitable. Project A would have had the same impact on my group, but it's a reflex to take points from others. I only invested in this project, I think it was the best compromise"
To harm the other group:	"This makes the others lose points"
If you invested in project C, why did you do it?	
Not invested:	"I didn't invest, I am not a terrorist!"
To harm the other group:	"When I invested in project C, it was only to reduce earnings of the blue group. (I don't know why I wanted that but I did want it)<br>"To sink the blues"
Was testing:	"I tested once or twice, investing few amounts to see the effect that it would make on the loses of points of the other team"

Table S5. Inter-rater agreement about classification of motives to invest in available projects

	Agreement	E(Agreement)	Kappa	Std. Err.	Z	Prob$>Z$
Project A	68.75%	24.00 %	0.5888	0.0738	7.97	0.0000
Project B	87.50%	20.36%	0.8431	0.0679	12.42	0.0000
Project C	93.75%	44.88%	0.8866	0.0972	9.12	0.0000

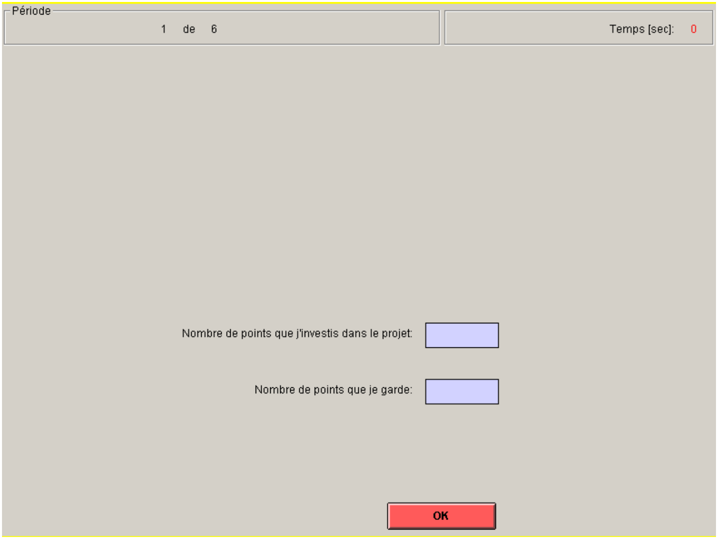

Figure S1. (text in Figure) Number of points that I invest in the project. Number of points that I keep.

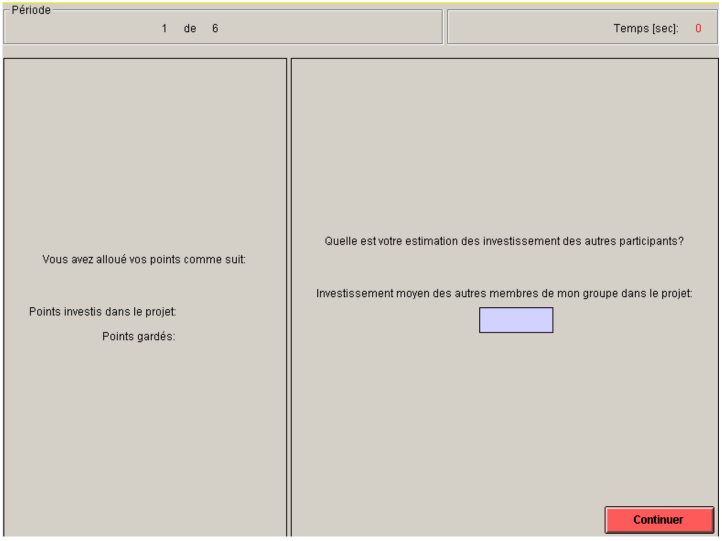

Figure S2. (text in Figure) LEFT: You have allocated your points as follows: points invested in the project; points kept. RIGHT: What is your estimation of the investments of the other participants? Average investment of the other members of my group in the project.

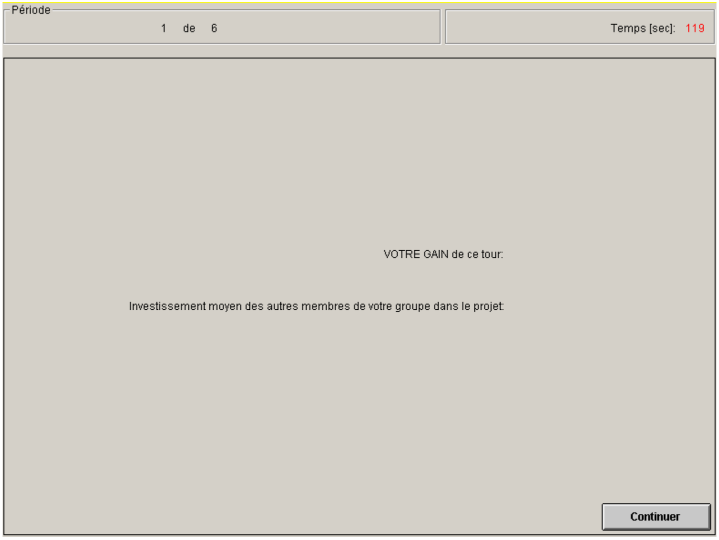

Figure S3. (text in Figure) Your earnings in this round. Average investment of the other members of your group in the project.

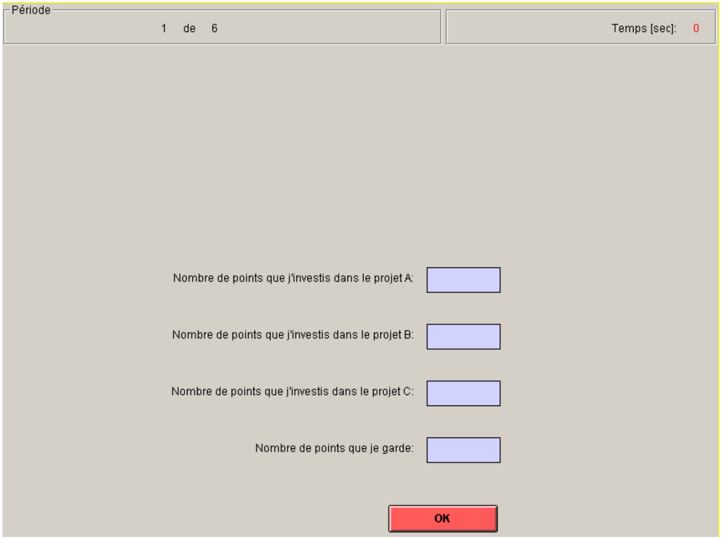

Figure S4. (text in Figure) Number of points that I invest in project A; B; C. Number of points that I keep.

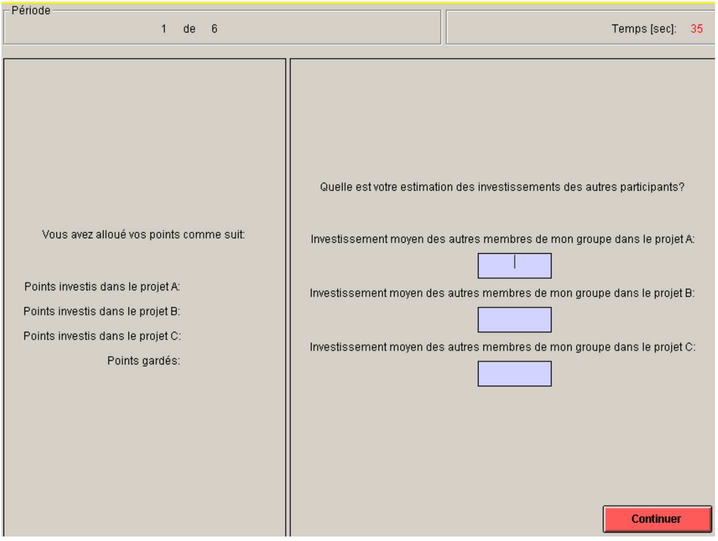

Figure S5. (text in Figure) LEFT: You have allocated your points as follows: points invested in project A; B; C; points kept. RIGHT: What is your estimation of the investments of the other participants? Average investment of the other members of my group in project A; B; C.

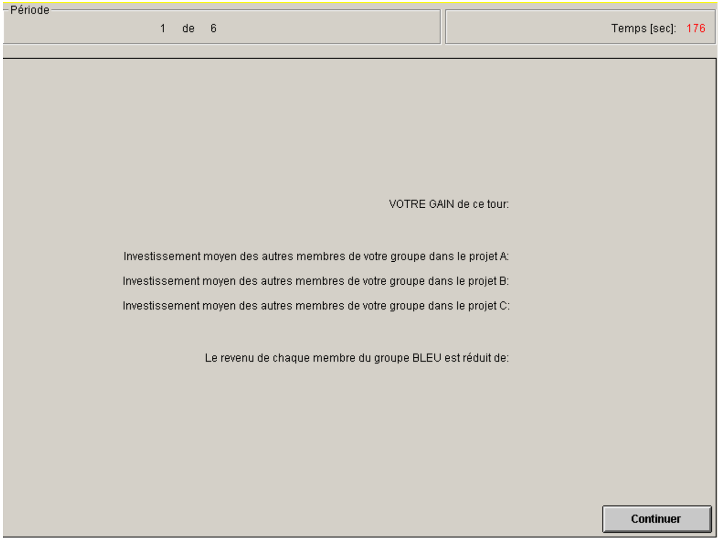

Figure S6. (text in Figure) Your earnings in this round. Average investment of the other members of your group in project A; B; C. The earnings of each member of the BLUE group are reduced by.

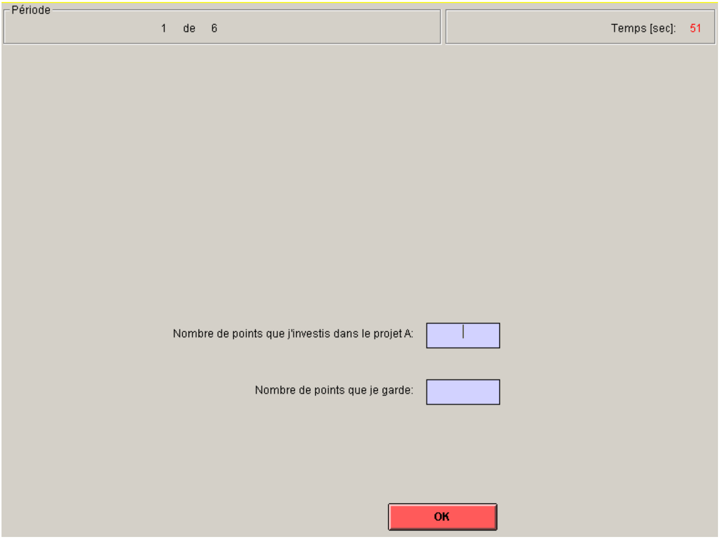

Figure S7. (text in Figure) Number of points that I invest in project A. Number of points that I keep.

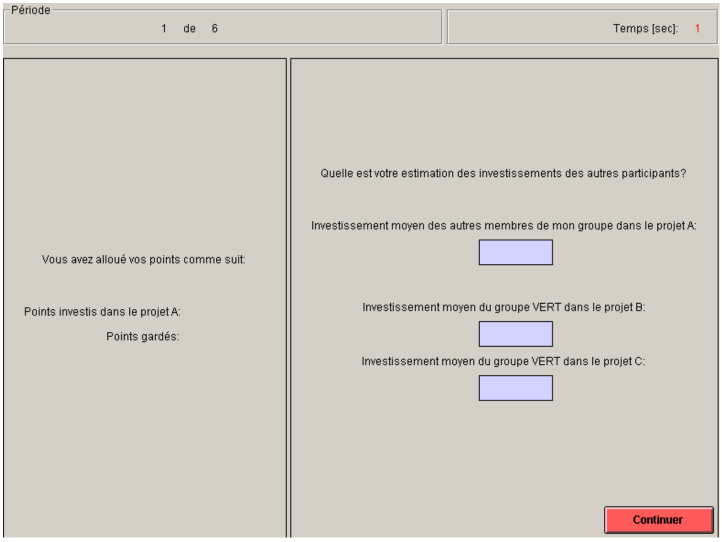

Figure S8. (text in Figure) LEFT: You have allocated your points as follows: points invested in project A; points kept. RIGHT: What is your estimation of the investments of the other participants? Average investment of the other members of my group in project A. Average investment of the GREEN group in project B; C.

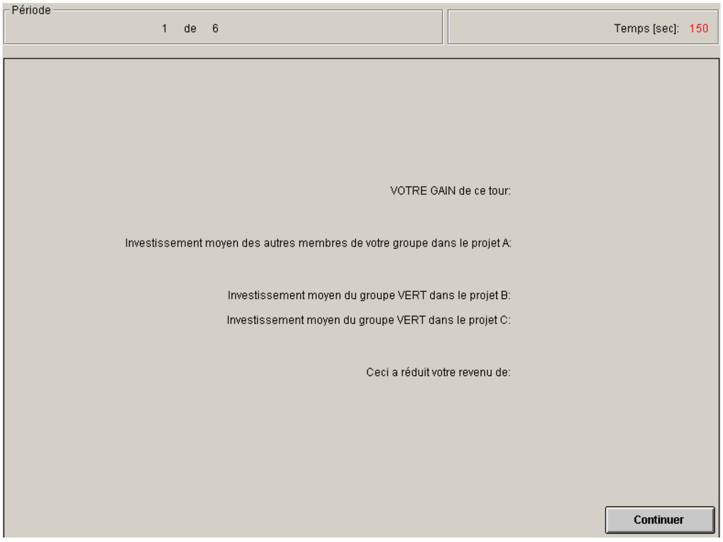

Figure S9. (text in Figure) Your earnings in this round. Average investment of the other members of your group in project A. Average investment of the GREEN group in project B; C. This has reduced your earnings by.
